# Maternal Injectable Mineral Administration Effects on Calf Growth and Reproductive Parameters

**DOI:** 10.3390/ani15030330

**Published:** 2025-01-24

**Authors:** Makayla A. Brenner, Rodrigo S. Marques, Christian J. Posbergh, Abigail L. Zezeski, Thomas W. Geary, Sarah R. McCoski

**Affiliations:** 1 Department of Animal and Range Sciences, Montana State University, Bozeman, MT 59717, USA; makayla.ogg@student.montana.edu (M.A.B.); christian.posbergh@montana.edu (C.J.P.); abby.zezeski@usda.gov (A.L.Z.); 2 School of Animal Sciences, Virginia Polytechnic Institute and State University, Blacksburg, VA 24060, USA; marques@vt.edu; 3 Fort Keogh Livestock and Range Research Laboratory, USDA-ARS, Miles City, MT 59301, USA; tom.geary@usda.gov

**Keywords:** bovine, developmental programming, puberty, trace minerals, sperm quality

## Abstract

Limited trace mineral research has focused on the impacts of maternal trace mineral supplementation on offspring fertility. We hypothesized that maternal injectable mineral (INJ) administration during mid/late gestation would improve overall growth and reproductive parameters in offspring. Two-hundred and seventy-eight cows were enrolled in the study and blocked by body weight, parity, and day (d) of gestation, and randomly assigned to a treatment group: a group (MM, *n* = 138) that received a single INJ (Cu = 15 mg/mL, Mn = 10 mg/mL, Se = 5 mg/mL, and Zn = 60 mg/mL; Axiota, Ft. Collins, CO, USA) or control group (CON, *n* = 138), which did not. Following calving, adjusted birth weight and weaning weights were recorded. A subset of bulls was developed until one year of age (CON, *n* = 18; MM, *n* = 14) when two semen ejaculates were collected for sperm quality. Mineral injection calves were heavier at birth than CON (*p* = 0.05). A treatment by parity interaction was observed for an increase in progressive motility in MM bulls than CON (*p* = 0.10). Injectable mineral administration to the pregnant multiparous cow improved sperm progressive motility in bulls, but no improvements were observed when assessed via flow cytometry.

## Simple Summary

Trace mineral developmental programming has become a popular area of research. The primary focus of this research is related to the role of trace minerals in calf growth until weaning, calf health, and feedlot growth and performance. Recently, there has been a focus on investigating the effects on heifer calf reproductive parameters; however, impacts on bull reproductive parameters remain under investigation. This study explored the role of maternal injectable trace minerals on calf growth and offspring reproductive parameters. Bulls whose dams received injectable trace minerals during gestation had increased body weights and minor improvements in bull fertility compared to their control counterparts. The current study suggests injectable mineral administration to the pregnant cow during mid and late gestation benefits bull offspring reproductive parameters and development prior to the start of the first breeding season.

## 1. Introduction

Montana is the largest seedstock-producing state in the United States, highlighting the importance of reproductive efficiency in both males and females as a critical component to the success of the operation [1]. It is important for heifers to become pregnant early in the first and subsequent breeding seasons, highlighting the importance of heifers reaching puberty prior to the breeding season [1,2]. Additionally, it is just as important for yearling bulls to reach puberty prior to the first breeding season to allow for maximum reproductive opportunities [3]. Previous work demonstrated the impacts of maternal nutrition on the reproductive function of offspring [4,5,6,7,8,9,10]; thus, the potential to improve beef herd productivity through developmental programming warrants further research to determine the impacts on fertility in the offspring.

Reproductive organ development occurs during early gestation [11], and ovarian follicle formation begins in mid-gestation and continues until birth [12]. Female offspring are born with a predetermined number of ovarian follicles [13]. Harvey et al. [10] reported that heifers from dams that received organic trace minerals (TM) during mid and late gestation reached puberty at an earlier age when compared to heifers from cows supplemented with sulfate sources of TM. The authors suggested the differences in attainment of puberty were due to the bioavailability of the organic sources of minerals to the dam, which promoted primordial follicle development [10].

Less research has investigated the effects of maternal nutrition during gestation on male reproductive organ development and spermatogenesis [8,9,14]. Much of this work focuses on protein supplementation or nutrient restriction [9,14]. Despite the importance of TM to male reproductive performance [15,16], the understanding of the effects of TM supplementation to the dam on male offspring reproductive performance is limited [8]. Maternal selenium (Se) supplementation during gestation affected the expression of genes associated with spermatogenesis and hormone production in the neonatal bovine testis [8]. The work by Cerny et al. [8] suggests that male bovine fetal testes are sensitive to maternal mineral nutrition while in utero; however, it is unknown how these changes in gene expression affect ejaculate concentration and quality.

Although the fetus relies on the dam for nutrients, including TM, for proper growth and development, it is relatively unknown how maternal TM supplementation impacts the reproductive development of the offspring. Previous work has acknowledged the importance of TM for fetal immune, nervous, and reproductive system development [17,18]. However, recent work [8,10,19] has only evaluated the effects of free-choice TM on offspring reproduction. The administration of injectable TM (INJ) is an advantageous supplementation strategy compared to free-choice TM supplementation because a known quantity of minerals is administered. Intake is variable with free-choice TM. Additionally, INJ TM has greater bioavailability to the animal because it is administered subcutaneously and bypasses the rumen, and it is quickly absorbed and used or stored by the animal [20]. Thus, it is possible that INJ has greater benefits to the fetal reproductive system. Thus, we hypothesized that offspring from dams administered a mineral injection would have improved reproductive parameters and overall growth. Our main objectives were to evaluate the effects of maternal mineral treatment on offspring: (1) growth until weaning, (2) ovarian development, and (3) peripubertal sperm characteristics.

## 2. Materials and Methods

This experiment was conducted at the USDA-ARS Fort Keogh Livestock and Range Research Laboratory (Miles City, MT, USA). All experimental procedures and protocols were approved by the Animal Care and Use Committee of Montana State University (#2022-261-AA).

### 2.1. Cow Management

Two-hundred and seventy-eight CGC composite (½ Red Angus, ¼ Charolais, ¼ Tarentaise) cows were blocked by body weight (BW; CON = 459 ± 140 kg; MM = 462 ± 153 kg), day (d) of gestation, and age (2–9 yrs. old), and randomly assigned to one of two treatment groups. All cows had access to free-choice TM (Vitaferm Conserve^®^, BioZyme Incorporated, Saint Joseph, MO, USA) for a minimum of 90 d prior to the start of the study. The treatments were as follows: (1) control cows (CON, *n* = 139) received no further treatment, and (2) mineral injection cows (MM, *n* = 139) were administered a single subcutaneous INJ (copper (Cu) = 15 mg/mL, manganese (Mn) = 10 mg/mL, Se = 5 mg/mL, and zinc (Zn) = 60 mg/mL; Axiota, Ft. Collins, CO, USA; approximately 1 mL/90 kg BW according to label) on d 182 ± 1.04 of gestation. All cows, regardless of treatment, received one of two different TM packages based on the timing of the production cycle (Vitaferm Conserve or Vitaferm ConceptAid 5/S, BioZyme Incorporated). Additionally, all cows grazed warm and cool season forages. The forages at Fort Keogh Livestock and Range Research Laboratory have known mineral deficiencies, as reported by Grings et al. [21]. Thus, all cows were provided a commercially available free-choice TM package (Vitaferm Conserve and Vitaferm ConceptAid 5/S, BioZyme Incorporated) for a minimum of 90 d prior to the start of the study.

### 2.2. Pre-Weaning Management

All cows were managed as a single group from calving until weaning, but primiparous cows were pastured separately from multiparous cows before calving. At calving, calf sex (CON, females = 64 and males = 71; MM, females = 69 and males = 66) and birth weight was recorded. Adjusted birth weights were calculated using the Beef Improvement Federation [22] guidelines [23]. Calf weights were recorded at approximately 166 d of age (preconditioning) and 195.5 d of age (weaning). Adjusted 205-day weights and average daily gain (ADG) were calculated based on the BIF [23] guidelines adjusting for calf sex and dam age.

### 2.3. Replacement Heifer Management

Following weaning, a subset of heifers was retained as replacement heifers (*n* = 27; CON, *n* = 12; MM, *n* = 15). All heifers were managed as a single group and were provided a total mixed ration consisting of TM (Vitaferm ConceptAid 5/S, BioZyme Incorporated), corn, corn silage, and hay to satiety. A diet nutrient analysis was completed by Midwest Laboratories (Omaha, NE, USA; Table 1). Heifer weights were recorded at approximately 228, 264, 320, 349, 382, and 413 d of age.

Beginning at approximately 10 months of age, blood samples were collected via coccygeal vasculature at 9–11 d intervals until approximately 14 months of age. Blood was centrifuged at 2500× *g* for 30 min at 4 °C, and plasma was harvested and frozen at −20 °C until progesterone (P4) analysis. Plasma P4 concentrations were evaluated in duplicate by radioimmunoassay using a commercially available kit (MP Biomedical, Irvine, CA, USA) as reported previously [24]. At 14 months of age, ovaries were examined via transrectal ultrasonography (Aloka SSD 3500 V and 7.5 MHz convex transducer), and the follicle number on each ovary was recorded. Attainment of puberty was determined either when two consecutive plasma samples contained ≥1 ng/mL of P4 or when a corpus luteum was present on the ovary at 14 months of age and a single plasma sample with ≥1 ng/mL.

All heifers received a controlled internal drug release (CIDR; Eazi-Breed^TM^ CIDR^®^; Zoetis Inc., Kalamazoo, MI, USA) containing 1.38 gm of P4 at 14 months of age to assist with estrous synchronization. The CIDR was removed 5 d after insertion, and all heifers received 25 mg of dinoprost tromethamine (PGF; 5 mL, Lutalyse, Zoetis Inc.) and an estrus detection patch (Estrotect; Estrotect Inc., Spring Valley, WI). Trained personnel observed heifers for signs of estrus twice daily (a.m. and p.m.). Heifers with an activated estrus detection patch or that were observed in standing estrus by 60 h post PGF received artificial insemination (AI) at 60 h. The remaining heifers received AI and were administered 100 μg of gonadotropin-releasing hormone (2 mL, Factrel, Zoetis Inc.) coincident with AI at 96 h post PGF. Heifers were exposed to clean-up bulls beginning 9 d after the last AI for a 60 d breeding season. At 60 d after AI, pregnancy status was determined via transrectal ultrasonography using an Aloka SSD 3500 V and 7.5 MHz convex transducer. A second pregnancy diagnosis was conducted 90 d after AI using an Aloka SSD 550 V with a 5 MHz probe.

### 2.4. Bull Management

Following weaning, a subset of bulls (*n* = 32; CON, *n* = 18; MM, *n* = 14) was retained and developed to be used for breeding at one year of age. All bulls were managed as a single group and were provided a total mixed diet consisting of TM (Vitaferm ConceptAid 5/S, BioZyme Incorporated), corn, corn silage, and hay to satiety (Midwest Laboratories; Table 1). Bulls were weighed on 228, 264, 292, 312, 340, 375, and 396 ± 1 d of age.

#### 2.4.1. Semen Collection and Analysis

At 396 ± 1 d of age, two semen ejaculates were collected from bulls (*n* = 32; CON, *n* = 18; MM, *n* = 14. One cryptorchid bull (MM) was identified at the time of semen collection and was removed from further analyses. Scrotal circumference (SC) was collected from all bulls by one of two trained technicians using a tape measure. Semen samples were collected from bulls by trained technicians using a Pulsator IV electro-ejaculator (Lane Manufacturing Inc., Denver, CO, USA). All bulls were given a three-minute rest period between ejaculates, and a total of two ejaculates were collected from each bull. Ejaculates were maintained at 37 °C throughout all measurements. Sperm concentration was determined using the Accuread spectrophotometer (IMV Technologies, Maple Grove, MN, USA). Progressive motility (PM) was evaluated using the AndroScope CASA (Minitube, Verona, WI, USA) immediately following semen collection. A minimum of four fields containing at least 1000 sperm were evaluated. The sample with the greater PM chuteside was extended in OptiXcell (IMV Technologies) at a 1:1 (*v*:*v*) dilution and evaluated for functional sperm measures using flow cytometry (FC) approximately 3.5 h after collection.

#### 2.4.2. Morphology

Morphology slides were prepared in eosin/nigrosine morphology stain (Lane Manufacturing Inc., Denver, CO, USA) immediately after collection and evaluated under oil at 1000× magnification for 100 sperm on a BZ-X710 fluorescence microscope (Keyence, Itasca, IL, USA). Sperm were classified as either normal or abnormal, as previously described [25]. Recorded abnormalities included knobbed acrosomes, head defects, bowed mid-pieces, dag defects, distal midpiece reflex, coiled principal pieces, bent principal pieces, and distal droplets [25] as part of the bull breeding soundness evaluation (BBSE; [26]).

#### 2.4.3. Flow Cytometry Measurements

Upon arrival to the lab, all samples were washed using 10 mL of bovine non-capacitation media (bNCM; pH of 7.2, NaCl [100 mM], NaH2PO4 [0.3 mM], KCl [3.1 mM], MgCl_2_·6H_2_O [0.4 mM], polyvinyl alcohol [0.01 mM], Na-pyruvate [1 mM], Na-lactate [22 mM], HEPES [40 mM], gentamicin [0.1 mM], penicillin G [0.174 mM], and fructose [54 mM]) to remove the seminal plasma and extender. Samples were centrifuged at 500× *g* for 10 min at room temperature, the supernatant was discarded, and the pellet was resuspended in bNCM to 80 × 10^6^ sperm per mL. An aliquot was then diluted 1:1 (*v*:*v*) with bNCM to a final concentration of 40 × 10^6^ per mL for analysis. Samples were analyzed via FC using a Guava EasyCyte B/G/V-HT equipped with violet (405 nm, 110 mW), blue (488 m, 160 mW), and green (532 nm, 110 mW) lasers and with GuavaSoft-4.5 software (Cytek, Fremont, CA, USA). DNA integrity was analyzed with a Guava EasyCyte 5-HT equipped with a blue (488 nm, 160 mW) laser and GuavaSoft 1.0 software (IMV Technologies, L’Aigle, France). Populations of sperm were classified by forward and side scatter acquisition with a flow rate of less than 5000 cells/µL. Each assay included evaluation of >5000 events. All results from the resulting dot plots were recorded as percentages. Compensation was performed after acquisition when necessary. The flow cytometer was cleaned and calibrated daily using Guava easyCheck calibration beads (Cytek).

#### 2.4.4. Mitochondrial Membrane Potential/Viability

In a 96-well plate, 25 μL of 8 μM mitochondrial membrane potential dye JC-1 (8 μM) was added, followed by 10 μL of ethanol (200 proof), 163 μL of bNCM, 2 μL of 19 mg/mL viability dye calcein violet AM (CV), and 1 μL of sample. The plate was incubated for 40 min at 37 °C. The 405 nm laser was used for the excitation of JC-1 and CV, and acquisition occurred with a 450/45 nm detector for CV and a 620/52 nm detector for J-aggregate formation in spermatozoa [27,28]. Data were evaluated as the percentage of viable polarized or depolarized and non-viable polarized or depolarized sperm.

#### 2.4.5. Zinc Signatures, Viability, Acrosome Integrity and Capacitation

Samples were evaluated for Zn signatures, acrosome integrity, and viability to assess capacitation status. At time (T) 0, 25 μL of sample, 10 μL of 12.5μM FluoZin3-AM (FZ3), 7.5 μL of 1 mg/mL of PNA Alexa Fluor 594 (PNA594), 5 μL of 19 mg/mL of calcein violet AM, 1 μL of aqua fluorescent dye (AF), and 51.5 μL of bNCM were added to a 1.5 mL tube. Samples were incubated at 37 °C for 30 min in the dark. Samples were centrifuged at 310× *g* for 5 min, and the supernatant was discarded. The pellet was resuspended in 100 μL of bNCM, and samples were incubated for 15 min at 37 °C. Following incubation, 250 μL of bNCM and 6 μL of the sample were added to a 96-well plate for FC measurements. Sperm cells were sorted as viable, dead, and dying cells: dead sperm with intact acrosome, dead sperm with disrupted acrosome, viable sperm with intact acrosome, and viable sperm with disrupted acrosome. Viability was determined by CV (live cells, 405 nm ex./450 nm em.) and AF (dead cells, 405 nm ex./525 nm em.). A gate was placed around CV-positive and AF-positive cells to eliminate debris for acrosome integrity and Zn signature analysis. Quantification of acrosome disruption was determined by PNA594 (532 nm ex./525 nm em.) and AF. Sperm Zn signatures were evaluated using FZ3 (488 nm ex./525 nm em.) and AF and confirmed with fluorescence microscopy (described by Gonzalez-Berrios et al. [29]). Sperm were capacitated in bovine capacitation media (bCM, bNCM with the addition of 2.1 mM CaCl_2_ 2H_2_O, 25 mM NaHCO_3_, 10 μg/mL heparin, 0.6% BSA, pH 7.4, made at 2× concentration) to evaluate the stage of capacitation (Gonzalez-Berrios et al., 2024). An aliquot of each sample at 80 × 10^6^ per mL was diluted to 40 × 10^6^ in bCM 1:1 (*v*:*v*) and incubated at 37 °C until assessment of capacitation signatures 4 h later (T4). At T4, the same process as described above was completed. Motility was assessed via AndroScope CASA at T0 and T4.

#### 2.4.6. Reactive Oxygen Species

To evaluate the ability of sperm to withstand reactive oxygen species (ROS), samples were challenged with hydrogen peroxide (H_2_O_2_) as a measure of stress susceptibility. In a 1.5 mL tube, 190 μL of bNCM, 10 μL of 100 mM of dihydrorhodamine 123 (DHR123), and 2 μL of sperm were combined. After incubating for 20 min at 37 °C, 2 μL of H_2_O_2_ (0.12%) was added to each sample, mixed, and incubated at 37 °C for 40 min. Following incubation, 600 μL of bNCM was added, and the mixture was centrifuged at 310× *g* for 5 min. The supernatant was discarded, and the pellet was resuspended in 300 μL of bNCM. Samples were transferred to a 96-well plate, and 1 μL of 1 mg/mL propidium iodide (PI) was added. The 488 nm laser was used for the excitation of both DHR123 and PI. The 512/18 detector was used for DHR123 acquisition, and the 695/50 nm detector was used for PI acquisition. All sperm populations were measured as the percentage of viable or non-viable (PI−/+) with the ability to withstand ROS (ROS+) or not (ROS−).

#### 2.4.7. DNA Integrity

DNA damage was measured by flow cytometry using acridine orange, as previously reported by Evenson [30]. Briefly, 2 μL of sperm cells were added to 98 μL of TNE buffer (0.15 M NaCl, 0.01 M Tris, 1 mM EDTA, pH 7.4). Next, 200 μL of acid detergent (0.15 M NaCl, 0.1% Triton X-100, 0.08 N HCl, pH 1.21) was added to each sample. After exactly 30 s, 600 μL of acridine orange staining solution (0.15 M NaCl, 1 mM EDTA, 0.1 M citric acid, 0.2 M Na_2_HPO_4_, 0.006 mg/mL acridine orange, pH 6.0) was added. After 2 min and 30 s, samples were analyzed via FC. Excitation of acridine orange occurred via the 488 nm laser, and evaluation of intact and fragmented DNA was captured with 512/18 nm detector and 695/50 nm detector, respectively. The percentage of fragmented DNA was quantified using easySoft 1.0 (IMV Technologies).

### 2.5. Statistics

All variables were analyzed with cow as the experimental unit and cow (treatment) as the random variable. All statistics were conducted in R Studio version 2023.09.01 Build 494 [31,32]. All results are reported as least square means (mean ± standard error).

#### 2.5.1. Pre-Weaning

All data were analyzed for normality using the Shapiro–Wilkes Test [33]. The gestation interval at the time of treatment (d 182 ± 1.04 of gestation) was classified as one of three intervals: (1) d 126 to d 156 of gestation (CON, *n* = 8; MM, *n* = 12); (2) d 157 to d 187 gestation (CON, *n* = 76; MM, *n* = 72); and (3) d 188 to d 219 of gestation (CON, *n* = 53; MM, *n* = 52). Treatment, gestation interval, and treatment x gestation interval were included in the model statements for adjusted birth weights, preconditioning BW, adjusted d 205 weaning weights, and ADG. All data were analyzed using a generalized linear model with Gaussian distribution. The least-square means (LSMeans) were calculated using the generalized linear model with Gaussian distribution and ANOVA with an F-statistic test to determine significance. Pairwise comparisons were used for all models to determine contrasts between treatment groups and gestation interval, as well as the corresponding interactions. Significance was set at *p* < 0.05, and tendency was set at *p* ≥ 0.05 and <0.10.

#### 2.5.2. Heifer

The initial models included treatment effect, gestation interval, cow parity (primiparous or multiparous), and the corresponding interactions. Gestation interval is described as either ≥d 184 (CON, *n* = 5; MM, *n* = 10) or <d 184 (CON, *n* = 7; MM, *n* = 5) of gestation. Data were analyzed for normality using the Shapiro–Wilkes test. Repeated variables were BW at 228, 264, 320, 349, 382, and 413 d of age. Treatment, day of age, gestation interval, cow parity, and the corresponding interactions were included in the model statements for BW and ADG. Heifer BW and ADG were analyzed using a generalized linear mixed model with a Gaussian distribution and ANOVA with an F-statistic test. Gestation interval and cow parity were removed from the model due to not being significant. Age at puberty and total number of follicles were analyzed as single points in time using a generalized linear model with Gaussian distribution and ANOVA with an F-statistic test to determine significance. Heifer pregnancy rates (pregnant or open) were analyzed as a binary variable using a generalized linear model with a binomial link logit function and ANOVA with the Chi-square statistic. Least square means and pairwise comparisons were used for all models to determine the contrasts between treatments. Both gestation interval and cow parity were removed from the age at puberty and AFC models because they were not significant. Gestation interval and cow parity were removed from the ADG model because they were not significant. Significance was set at *p* < 0.05, and tendency was set at *p* ≥ 0.05 and <0.10.

#### 2.5.3. Bull

Gestation interval was described as either ≥d 185 of gestation (CON, *n* = 8; MM, *n* = 8) or <d 185 of gestation (CON, *n* = 10; MM, *n* = 6) as a binary variable. Cow parity (primiparous or multiparous) was also described as a binary variable. Treatment, gestation interval, cow parity, treatment x gestation interval, or treatment x cow parity were included in all models. Repeated variables were BW (approximately monthly from weaning to the day of BBSE) and ADG. Bull BW and ADG were analyzed using a generalized linear model with a Gaussian distribution and ANOVA with an F-statistic test to determine significance. The least-square means and pairwise comparisons were used for all models to determine the contrasts between treatment groups, cow parity, gestation interval, day, and the corresponding interactions. All the initial models included gestation interval and cow parity at the time of treatment administration and were removed from the model when not significant.

Scrotal circumference; sperm PM; ejaculate concentration; mitochondrial membrane potential; DNA integrity; ROS; sperm morphology; PM at T0 and T4; and Zn signatures were analyzed at single time points. Flow cytometry measurements for ROS, Zn signatures, DNA integrity, and mitochondrial membrane potential were beta-distributed, and the link logit function and ANOVA with the F-statistic were used to analyze the data. Progressive motility at the time of collection, T0, and T4 (both bNCM and bCM) were analyzed using the beta regression model with the link logit function and the ANOVA with the F statistic. Sperm concentration at collection and SC were analyzed using a linear mixed model. The least-square means and pairwise comparisons were used for all models to determine the contrasts between treatment groups, cow parity, gestation interval, and the corresponding interactions. Gestation interval and cow age were included in all the initial models and were removed when not significant. Significance was set at *p* < 0.05, and tendency was set at *p* ≥ 0.05 and <0.10.

## 3. Results

### 3.1. Pre-Weaning Development

Birth weights were heavier (*p* = 0.05) for calves born to MM than CON cows (Table 2). Regardless of treatment, there was an effect of gestation interval on calf birth weight (*p* < 0.0001), with calves born earlier in the season being heavier than calves born later in the season (Table 2). A tendency for an increase in adjusted d 205 weights was observed, where MM calves tended to be heavier and have a greater ADG than CON at weaning (*p* = 0.07; Table 2). Additionally, there was an effect of gestation interval on calf ADG between birth and weaning (*p* < 0.001), with younger calves having a greater ADG compared to older calves (Table 2).

### 3.2. Replacement Heifer Development

A day effect was observed for heifer BW (CON = 275 ± 2.33 kg, MM = 275 ± 2.10 kg; *p* < 0.001) and ADG (CON = 0.39 ± 0.02 kg/d and MM = 0.43 ± 0.19 kg/d; *p* < 0.001). However, no differences were observed between treatments for heifer BW or ADG during the postweaning period (*p* ≥ 0.14). No differences were observed in the total number of follicles (CON, *n* = 10.4 ± 1.36 vs. MM, *n* = 11.8 ± 0.90; *p* = 0.30) or age at puberty between the MM (356 ± 41.5 d of age) and CON heifers (391 ± 27.6 d of age; *p* = 0.49). Heifer pregnancy rates were not different between MM and CON heifers and were not affected by the gestation interval of INJ treatment in utero (CON = 83.3 ± 0.1% and MM = 86.7 ± 0.8%; *p* > 0.10).

### 3.3. Bull Development

**Figure 1:**
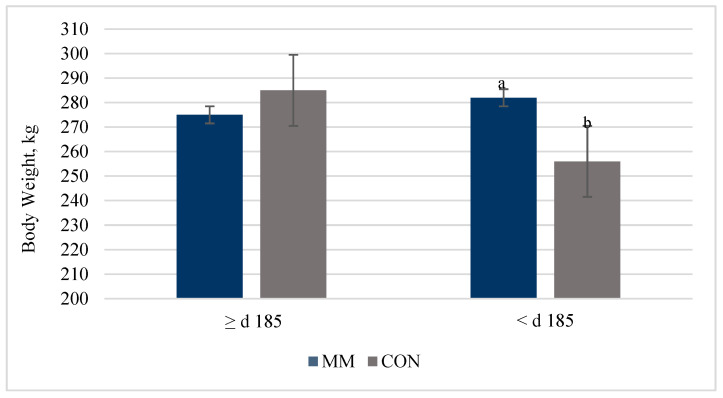
A treatment by day of gestation interval was observed (*p* < 0.0001) for bull BW postweaning. Mineral injection (MM) bulls that were < d 185 of gestation at the time of treatment tended to be heavier than CON. ^a,b^ superscripts that differ between means indicate a *p* < 0.0001.

Bulls whose dams received INJ during gestation tended to be heavier during the postweaning period than CON bulls (*p* = 0.06). Additionally, there was a gestation interval effect (*p* < 0.0001) and a day effect (*p* < 0.0001) for bull BW to increase during the postweaning period. There was a treatment by gestation interval interaction for bull BW throughout the postweaning period (*p* < 0.0001; Figure 1).

Bulls from cows that were <d 185 of gestation when INJ was administered were heavier during the study compared to CON bulls of a similar age (Figure 1). No differences were observed for ADG between treatment groups postweaning (*p* = 0.28).

### 3.4. Bull Fertility Measures

No differences were observed in SC between bulls whose dams received INJ during gestation compared to bulls from CON dams (*p* > 0.10). Gestation interval affected SC (*p* = 0.01; Table 3), indicating older bulls had larger SC. A treatment by gestation interval interaction existed for SC (*p* = 0.03, Table 3). The older CON bulls had a larger SC (*p* = 0.03) compared to the younger CON bulls (Table 3). The percentage of normal sperm in the ejaculate was not affected (*p* = 0.14) by the main effect of treatment in utero but tended (*p* = 0.09) to be affected by treatment by gestation interval (Table 3). Bulls that received INJ during later gestation (68.9 ± 5.13%) tended to have a greater percentage of normal sperm in the ejaculate than younger MM bulls (48.8 ± 5.92%; *p* = 0.07; Table 3). There were no differences (*p* ≥ 0.20) in capacitation signatures at T0 or T4 between bulls that received INJ in utero and CON bulls.

**Figure 2:**
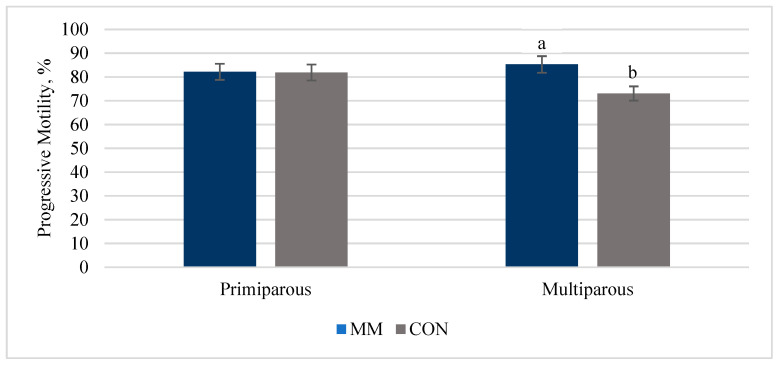
A treatment by cow parity (*p* = 0.10) was observed for progressive motility at the time of breeding soundness examinations. Mineral injection bulls from multiparous cows had greater (85.3 ± 5.0%) progressive motility compared to CON bulls (73.1 ± 5.0%) whose multiparous dams had access to free-choice trace minerals (*p* = 0.10). ^a,b^ superscripts that differ between means indicate a *p* = 0.10.

No differences were observed between MM and CON bulls for sperm PM at the time of semen collection (*p* = 0.29). Bulls born to primiparous dams tended to have sperm with greater PM (*p* = 0.09) than bulls born to multiparous dams (Figure 2). A tendency for treatment by cow parity interaction was observed for sperm PM at the time of semen collection (*p* = 0.10; Figure 2). Mineral injection bulls (85.3 ± 4.19%) born to multiparous cows tended to have sperm with greater PM (*p* = 0.10; Figure 2) compared to CON bulls born to multiparous cows (73.1 ± 4.19%).

At the time of semen collection, no differences were observed in the concentration of sperm in the ejaculate between MM and CON bulls (*p* = 0.31; Table 4). Additionally, no differences were observed between the MM and CON bulls for the percentage of viable sperm, viable sperm with intact acrosome, viable sperm with polarized mitochondria, viable sperm with ROS+, or integrity of DNA of sperm (*p* ≥ 0.28; Table 4).

## 4. Discussion

Multiple studies, both from this group and others [19,34,35], have documented the transfer of trace minerals, including INJ, from the dam to the fetus during pregnancy (unpublished data from this group [34]). Previous work from this group (unpublished) and Stokes et al. [34] indicate benefits from the INJ to both the pregnant cow or heifer and neonatal calf. The work by Stokes et al. [34] documented changes in circulating and hepatic mineral concentrations of the pregnant animal and their offspring; thus, the current study did not evaluate mineral concentrations, as it has been documented in the previous literature. Therefore, the goal of this study was to determine a period of gestation that is ideal for administering INJ and can be used to improve reproductive parameters in the offspring. This is the first study to the authors’ knowledge that aims to identify the ideal time during gestation to administer INJ to improve the weaning weights of calves and is the first to evaluate semen quality and sperm viability in male offspring.

### 4.1. Pre-Weaning Development

Calves whose dams received INJ had an increase in adjusted birth weight and tended to have an increase in adjusted d 205 weights and ADG between birth and weaning compared to CON. Our results differ from those of previously published work, where authors did not report an increase in birth weights when dams received TM during gestation [34,35,36]. Alternatively, Stokes et al. [34] reported a decrease in calf birth weight when injectable trace mineral was administered during early, mid, and late gestation. In this study, administration of INJ, regardless of gestation interval, improved calf growth in utero and tended to improve growth until weaning. Therefore, administering INJ to the pregnant cow may have greater economic benefits to the producer when marketing calves at weaning. The gestation range of cows in the current study when MM was administered was d 126 to 219, which may have influenced results.

### 4.2. Heifer Development

No differences were observed in the age of puberty or total number of follicles present between MM and CON heifers. These results are in agreement with Hurlbert et al. [19], who also did not observe differences in the age of puberty or the number of follicles when heifers were supplemented with vitamins and TM throughout gestation. Conversely, Harvey et al. [10] reported earlier attainment of puberty among heifers whose dams received organic TM from d 117 of gestation until calving. These differences may be due to the timing of INJ administration to the pregnant cow, sample size, or both. In the current study, all cows received free-choice TM, and it was not until mid or late gestation (d 126 to 219) that INJ was administered. Around d 90 of gestation, primordial follicle development begins [11], and the ovary may be more responsive to dietary changes and mineral treatments earlier in gestation compared to late gestation. Another possible explanation for the differences between the current study and Harvey et al. [10] is the number of heifers that attained puberty. In the current study, seven heifers (CON, *n* = 4 and MM, *n* = 3) out of the 27 heifers attained puberty, whereas Harvey et al. [10] reported that all heifers reached puberty. The limited number of pubertal heifers in the current study limits the interpretation of INJ effects on the age of puberty. This may have impacted the AI pregnancy rates between the CON and MM heifers, which were similar. These results are similar to those reported by Harvey et al. [10]. The current study did not have sufficient power to draw valuable conclusions pertaining to heifer reproductive parameters, and future studies should include a greater number of animals.

### 4.3. Bull Development

When pregnant cows were administered INJ before d 185 of gestation, bull offspring had an increase in BW postweaning compared to CON bulls. Marques et al. [35] observed an increase in BW of steers at the end of the feedlot period when cows were supplemented with organic TM 200% over the NASEM (2016) recommendations compared to a control group that did not receive TM. However, other groups [10] have reported no differences in steer performance at the end of the feedlot period when cows received inorganic TM. The weight advantage in the current study is likely a result of the increase in adjusted birth and the tendency for increased d 205 weaning weights in MM calves. Injectable mineral administration to the pregnant cow before d 185 of gestation positively impacts the male fetus, resulting in improved fetal and postnatal growth throughout the study.

Progressive motility of sperm at the time of semen collection was increased in MM bulls born to multiparous cows compared to CON bulls born to multiparous cows. This observed decrease in PM among CON bulls born to multiparous cows could have been due to the limited number of observations, and caution is urged in this interpretation until this finding is repeated in a more robust sample size. Regardless, this increase in PM did not translate into improvements in other sperm functional parameters when evaluated by FC. In conclusion, the treatment by cow parity interaction suggests the administration of INJ to the multiparous cow may improve sperm PM of bulls. Table 5 is included here to illustrate the foundational capacitation signatures of yearling bulls and the progression of sperm through capacitation signatures during 4 h of capacitation.

## 5. Conclusions

In summary, maternal INJ administration during mid to late gestation increased calf birth weights and tended to increase weaning weights and ADG between birth and weaning. Additionally, there were no improvements in heifer body weight postweaning or reproductive parameters. However, bulls born to dams receiving INJ during gestation tended to demonstrate an increase in postweaning growth. In addition, bulls born to multiparous dams that received INJ during gestation had an increase in sperm PM among ejaculates collected at approximately 14 months of age. However, INJ treatment of dams did not translate into improved sperm functional measures based on FC evaluation. The ideal time (gestation interval) to administer INJ to pregnant cows to observe improvements in reproductive measures of offspring will require additional studies as we report no difference in heifer subsequent reproductive measures and conflicting minor improvements in bull fertility. It is possible that the differences observed in this study are due to an increase in sensitivity of the fetal testes compared to the fetal ovaries during in utero development, and future studies should investigate potential mechanisms causing the conflicting results observed in the current study.

## Figures and Tables

**Table 1:** Nutrient analysis of the total mixed ration on a dry matter basis that was fed to the CON and MM bulls and heifers during the development period.

	Bull Diet	Heifer Diet
Dry matter, %	86.90	87.41
Crude protein, %	9.11	9.13
ADF, %	25.20	27.63
TDN, %	69.60	67.75
NEL, Mcal/kg	0.72	0.68
NEM, Mcal/kg	0.70	0.68
NEG, Mcal/kg	0.43	0.41
S, %	0.12	0.13
P, %	0.25	0.24
K, %	1.13	1.21
Mg, %	0.22	0.24
Ca, %	0.35	0.38
Na, %	0.01	0.01
Fe, ppm	119.15	135.92
Mn, ppm	29.10	32.32
Cu, ppm	5.75	6.30
Zn, ppm	30.73	32.30

Ca = calcium; P = phosphorus; NaCl = sodium chloride; Mg = magnesium; K = potassium; Co = cobalt; Cu = copper; I = iodine; Mn = manganese; Se = selenium; Zn = zinc; % = percentage; ppm = parts per million; IU/kg = international units per kilogram; Mcal/kg = megacalories per kilogram. The bull diet consisted of 20% corn, 60% corn silage, and 20% hay. The heifer diet consisted of 10% corn, 70% corn silage, and 20% hay. Analyzed by Midwest Laboratories (Omaha, NE, USA). Supplemented to the replacement heifers and bulls from d 299 until the study was completed.

**Table 2:** Adjusted birth weight, adjusted d 205 weight, and preweaning average daily gain of calves whose dams received a single mineral injection (MM) or not (CON) during gestation.

	TRT			Gestation Interval				TRT x Gestation Interval									*p*		
								d 126–156			d 157–187			d 188–219					
	CON	MM	SEM	d 126–156	d 157–187	d 188–219	SEM	CON	MM	SEM	CON	MM	SEM	CON	MM	SEM	TRT	Gestation Interval	TRT x Gestation Interval
Adjusted birth weight, kg	34.6	35.9	0.85	34.6 ^a^	34.4 ^a^	36.8 ^b^	1.11	32.4	36.8	2.22	34.3	34.6	0.80	37.2	36.4	0.96	0.05	<0.001	0.10
Adjusted d 205 WW, kg	207	213	3.75	209	212	210	5.22	200	218	9.84	211	213	3.50	211	209	4.20	0.07	0.41	0.19
ADG, kg/d	0.96	0.99	0.02	1.14 ^a^	0.97 ^b^	0.83 ^c^	0.02	1.08	1.18	0.05	0.96	0.98	0.02	0.83	0.83	0.02	0.07	<0.001	0.24

Mineral injection (MM; 5 mL for 2-year-old cows (primiparous) and 6 mL for 3–9-year-old cows (multiparous); Cu = 15 mg/mL, Mn = 10 mg/mL, Se = 5 mg/mL, and Zn = 60 mg/mL; Axiota, Ft. Collins, CO, USA). SEM = standard error means. ^a,b,c^ superscripts differ between the means within the gestation interval column, indicating *p* < 0.001.

**Table 3:** Scrotal circumference and sperm morphology in bulls whose dams received a single mineral injection (MM) during gestation and had access to free-choice trace minerals or whose dams had access to free-choice trace minerals (CON).

	TRT			Gestation Interval			TRT x Gestation Interval						*p*		
							≥d 185			<d 185					
	CON	MM	SEM	≥d 185	<d 185	SEM	CON	MM	SEM	CON	MM	SEM	TRT	Gestation Interval	TRT x Gestation Interval
SC, cm	30.6	31.4	0.53	31.6	30.1	0.53	32.1 ^a^	31.1	1.05	29.2 ^b^	31.7	1.08	0.38	0.01	0.03
Normal, %	57.2	58.9	5.21	63.5	52.5	5.21	58.1	68.9 ^c^	7.25	56.2	48.8 ^d^	7.49	0.14	0.78	0.09

Mineral injection (MM; 5 mL for 2-year-old cows (primiparous) and 6 mL for 3–9-year-old cows (multiparous); Cu = 15 mg/mL, Mn = 10 mg/mL, Se = 5 mg/mL, and Zn = 60 mg/mL; Axiota, Ft. Collins, CO, USA). SEM = standard error means. ^a,b^ superscripts differ between the means within the TRT x gestation interval column, indicating that older CON bulls have an increase in SC compared to younger CON bulls (*p* = 0.03). ^c,d^ superscripts differ between the means within the TRT x gestation interval column, indicating that older MM bulls tended to have an increase in normal sperm than younger MM bulls (*p* = 0.07).

**Table 4:** Sperm concentration and functionality measures of bulls whose dams received a single mineral injection (MM) during gestation or not (CON) as determined by flow cytometry.

	Treatment Group			*p*
	CON	MM	SEM	TRT
Ejaculate Concentration, ×10^6^	262.0	337.0	73.1	0.31
Viability, %	60.8	64.3	5.5	0.53
Viable and polarized mitochondria, %	55.7	58.6	6.17	0.63
Viable and ROS+, %	33.2	38.0	4.53	0.29
Viable with intact acrosome, %	16.2	14.4	2.98	0.54
DNA fragmentation, %	8.04	6.56	1.57	0.36

Mineral injection (MM; 5 mL for 2-year-old cows (primiparous) and 6 mL for 3–9-year-old cows (multiparous); Cu = 15 mg/mL, Mn = 10 mg/mL, Se = 5 mg/mL, and Zn = 60 mg/mL; Axiota, Ft. Collins, CO, USA). SEM = standard error means.

**Table 5:** Capacitation signatures (%) for all bulls at time 0 (T0) and 4 h later (T4) following exposure to bovine capacitation media for all bulls in this study.

Description	T0	T4	SEM
Signature 1	7.40	2.25	1.25
Signature 2	11.10	12.10	2.19
Signature 3 (dead)	20.30	15.80	2.1
Signature 3 + 5 (dead)	3.91	3.51	0.62
Signature 3 + 5 (live)	35.90	17.60	3.72
Signature 4 (dead)	20.50	52.20	3.69

SEM = standard error mean.

## Data Availability

Dataset available upon request from the authors.
